# Limonene Enantiomeric Ratios from Anthropogenic and Biogenic Emission Sources

**DOI:** 10.1021/acs.estlett.3c00794

**Published:** 2024-02-02

**Authors:** Shan Gu, Wentai Luo, Avisa Charmchi, Kevin J. McWhirter, Todd Rosenstiel, James Pankow, Celia L. Faiola

**Affiliations:** † Ecology and Evolutionary Biology, University of California Irvine, Irvine, California 92697, United States; ‡ Civil and Environmental Engineering, Portland State University, Portland, Oregon 97201, United States; § Chemistry, University of California Irvine, Irvine, California 92697, United States; ∥ Biology, Portland State University, Portland, Oregon 97201, United States

**Keywords:** enantiomeric composition, volatile chemical products, biogenic volatile organic compound emissions, urban air quality

## Abstract

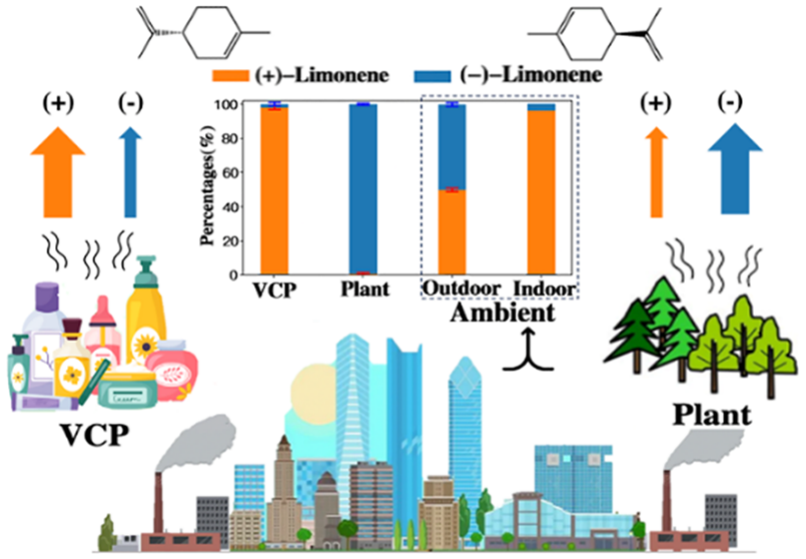

Emissions from volatile chemical products (VCPs) have been identified as contributors to air quality degradation in urban areas. Limonene can be a tracer compound for VCPs containing fragrances in densely populated regions, but limonene is also emitted from conifers that are planted in urban areas. This creates challenges for using limonene to estimate VCP emissions. In this study, the −/+ enantiomeric ratios of limonene from VCP and conifer emission sources were quantified to evaluate if this measurement could be used to aid in source apportionment and emission inventory development. Samples were analyzed using a gas chromatograph equipped with a chiral column and mass spectrometry. The results demonstrate that limonene exhibits distinct enantiomeric ratios when sourced from VCPs versus conifers. (+)-Limonene was dominant in VCP sources (>97%), which was not universally true for conifer sources. The results were compared to those of air samples collected outside at two locations and indoors. The levels of (−)-limonene in outdoor air in Irvine and Portland and in indoor air were 50%, 22%, and 4%, respectively. This suggests outdoor limonene had both VCP and plant emission sources while indoor air was dominated by VCP sources. This study demonstrates the potential utility of enantiomeric analysis for improving VCP emission estimates in urban areas.

## 1 Introduction

Air pollutants in Los Angeles and other urban areas continue to exceed regulatory limits, negatively impacting human and ecosystem health. The World Health Organization has identified indoor and outdoor air pollution as one of the top 10 causes of human mortality globally, leading to nearly 7 million premature deaths every year.^1^ In the past, transportation was a major source of this air pollution, along with industrial and residential sectors, and strict government regulation decreased the levels of these sources in Los Angeles, leading to substantial improvements in air quality.^2,3^ However, the downward trend in air pollution in Los Angeles has hit a plateau in recent years, particularly for ozone, which is difficult to regulate due to the nonlinear relationship between ozone production and its precursors (volatile organic compounds and nitrogen oxides).^4^ This stalling of progress suggests there are other unknown and/or understudied sources that also degrade air quality.^4,5^ In 2018, McDonald et al.^6^ presented evidence that volatile chemical products (VCPs) were a large underrepresented source of air pollution in Los Angeles. VCPs include chemicals in pesticides, coatings, printing inks, adhesives, cleaning agents, and personal care products. A follow-up study provided additional support and demonstrated that VCPs modulate urban air quality in densely populated regions like New York City.^7^ More and more evidence is showing that VCPs are important contributors to air quality degradation, and limonene has been identified as a molecular tracer for VCPs containing fragrances.^8^ However, limonene is also a major terpene emitted from many conifers and broad-leaf trees,^9^ and therefore assuming all urban limonene is derived from VCP sources likely leads to overestimation of the contribution of VCPs to air pollution. A more quantitative understanding of the anthropogenic (VCP) versus biogenic (urban trees) sources of limonene in urban areas is necessary to effectively mitigate air pollution issues.

Limonene is a common additive in shampoos, lotions, cleaners, etc. Limonene has two enantiomeric forms, (+)-limonene and (−)-limonene. Most limonene used in fragranced VCPs such as personal care products is derived from citrus peel waste, which contains a large proportion of limonene.^10^ Furthermore, the limonene in citrus peels is almost entirely the (+)-limonene enantiomer.^11−14^ The enantiomeric composition of limonene in non-citrus plants tends to be more variable between plant species. For example, the level of (+)-limonene can range from 21% to 71% in cannabis essential oil,^15^ range from 0% to 63% in pine essential oil,^16,17^ and reported as 68.5% in sage essential oil.^18^ In some plants, essential oils are dominated by the (−)-limonene enantiomer; just 0–6% of limonene in *Abies* species’ essential oils is (+)-limonene.^16^ In contrast to this interspecies variation, the enantiomeric composition of essential oils within a tree species appears to be stable, regardless of environmental context. For example, enantiomeric ratios of the chiral compounds present in pine essential oils were not altered by pathogen stress even though the composition of the oil was drastically changed after pathogen exposure.^19^ Note that these enantiomeric ratios have typically been reported in plant essential oils, not directly from the volatile organic compounds (VOCs) that are released to the atmosphere. Reports of limonene enantiomeric ratios in plant emissions are rare. One study showed that limonene in holm oak emissions is almost entirely the (−)-limonene form.^20^ Another recent study by Wang et al.^21^ presented enantiomeric ratios of monoterpene isomers emitted from six different conifers and found that the level of (−)-limonene ranged from <1% to >90%.^21^ Importantly, they also found that while the limonene enantiomeric ratio varied substantially between different tree species, intraspecies variation was low across individuals and over time, similar to what was observed in essential oils. Ultimately, while there is some expected variation in the limonene enantiomeric ratios from typical urban plants, there is less expected variation from personal care products, the latter of which should primarily contain (+)-limonene. Therefore, limonene enantiomeric analysis of ambient air could serve as a useful tool for establishing an upper limit for the contribution of VCPs to urban limonene and, with further method development that builds from this work, could be used to improve source apportionment of limonene in urban areas.

## 2 Materials and Methods

This study quantified limonene enantiomeric ratios from multiple personal care products (representing an anthropogenic VCP source) and conifers (representing a living biogenic source). These ratios were compared to limonene enantiomeric ratios in air samples collected at the University of California Irvine (UCI) and Portland State University (PSU). For the sake of clarity in our discussion, “anthropogenic limonene” refers to any limonene extracted from citrus peels and used in consumer products while “biogenic limonene” refers to limonene emitted directly by plants. We acknowledge that citrus essential oils are biogenic in origin, but emissions from fragranced VCPs that use citrus essential oils would not be considered “biogenic” from an emissions control perspective.

### 2.1 Emissions from Fragranced VCPs

Four personal care products were used in the study: a shampoo, a rose shower gel, a shower scrub, and a rose body lotion. Two milliliters of the product was placed in a gas chromatography (GC) vial, and the GC vial was placed in a mason jar equipped with inlet and outlet bulkhead unions on the lid. The jar was flushed with clean air for 20 min at a rate of 1 L/min. Clean air was generated with a zero-air generator (Environics, Inc.). Samples were collected from the headspace using adsorbent cartridges (Markes, TenaxTA, and Carbograph multibed stainless steel, Part C2-AXXXX-5149). Sample air was pulled through the cartridges at a rate of 0.15 L/min with a sampling pump (Sensidyne GilAir Plus) for 1–4 min depending on the rates of evaporation of different VCPs. Two sampling experiments were conducted for each product, and duplicate cartridges were used for each sampling event. Control samples were collected by using the same sampling procedure but with empty GC vials in the mason jar. Control samples confirmed that there was no limonene present when the GC vial was empty.

### 2.2 Emissions from Living Plants

Emissions from two tree species on the UCI campus were measured, including slash pine (*Pinus elliottii*) and loblolly pine (*Pinus taeda*). A dynamic branch enclosure technique was used to collect plant emission samples.^22^ Briefly, a 6 L oven roasting bag (ECOOPTS Inc., PET material) was placed around a branch and secured with a cable tie around the stem. The enclosure was purged for 20 min with 1 L/min of clean air to reach steady-state concentrations in the enclosure (3 times the residence time of the enclosure). Clean air was generated by passing ambient air through activated charcoal with an air pump (Sensidyne Inc., model BP120). The headspace from the branch enclosure was pulled through a multibed adsorbent cartridge using the same technique described in section 2.1. Duplicate cartridges were collected from each branch enclosure for each sampling event, and two sampling events were completed from the same branch for each tree species. Field blank control samples were collected by using the same sampling procedure without a branch inside the enclosure. Control samples confirmed that there was no limonene present in the empty enclosure.

### 2.3 Air Sampling

Twenty-four hour duplicate air samples were collected every other day on the rooftop of Steinhaus Hall on the UCI campus from July 19, 2023, to July 25, 2023 (total of 5 duplicate samples = 10 cartridges). The sampling setup consisted of an air pump (Sensidyne Inc., model BP120) and two Swagelok needle valves for controlling the rate of flow of air through the two cartridges. The sampling flow rate for each cartridge was set at 1 L/min. On September 6, 2023, additional air samples were collected at PSU in the first-floor lobby inside the Engineering Building (EB) and at an urban park (Park Blocks, situated between Millar Library and Fariborz Maseeh Hall) on the PSU campus. The sampling setup consisted of an air pump (MOA-V138-AA, Gast Manufacturing, Benton Harbor, MI) and a calibrated flow meter. The air was pulled through the cartridge at a flow rate of 2 L/min for 15 min.

### 2.4 Calculations

One of the duplicate cartridge samples collected at UCI for each type of sample was analyzed on site using a thermal desorption gas chromatograph–mass spectrometer (TD-GC-MS, Markes International TD-100xr autosampler, Agilent GC 7890 B, equipped with a 30 m DB-5 column, and Agilent 5975 MS). This analysis was conducted before the samples were sent to Portland to ensure they had a minimum of 10 ng for the enantiomeric quantification, which is a strong and easily quantifiable signal for the chiral analysis instrument. A more complete discussion of the instrument sensitivity and ambient detection limits is included in the section S1 of the Supporting Information. Relative emission profiles from each sample type were calculated by using the normalized peak area of each compound. Limonene enantiomeric ratios of cartridge samples were analyzed at PSU using a TurboMatrix 650 ATD (PerkinElmer Inc., Waltham, MA) unit interfaced to a Leco Pegasus 4D GC×GC-TOFMS (Leco Corp., St. Joseph, MI) running in 1-D GC mode (TOFMS, time-of-flight mass spectrometer). Additional description of the analytical method, standards, and uncertainty is included in the Supporting Information.

## 3 Results and Discussion

### 3.1 Composition of Emission Samples

**Figure 1:**
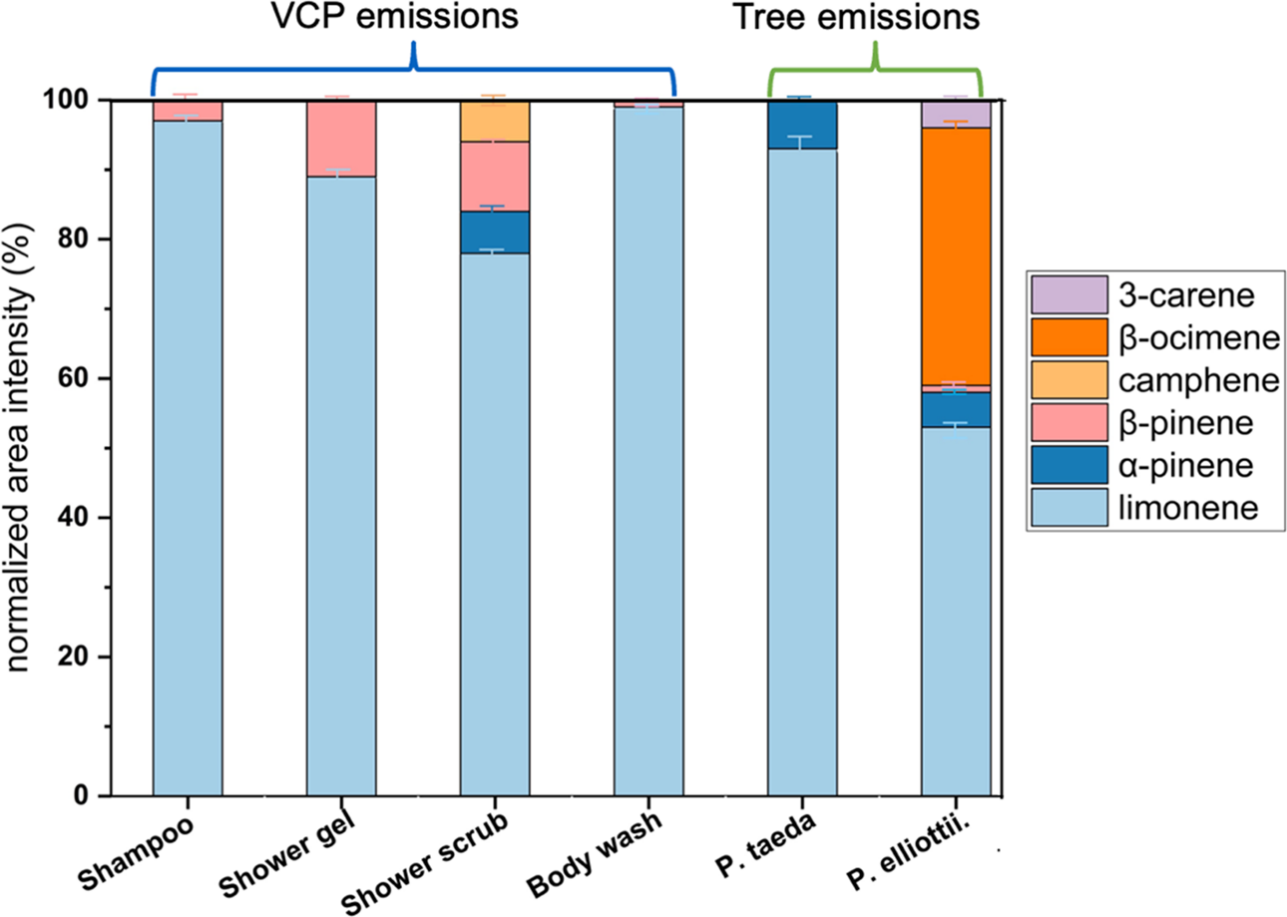
Relative contribution to mass spectrometer signal intensity for monoterpenes from VCP and living plant emission sources that were included in this study. All sources used in this study had substantial contributions of limonene in the cartridge samples. Error bars represent the standard deviation from duplicate cartridges.

The major monoterpenes emitted by all anthropogenic and biogenic sources evaluated in this study included limonene, 3-carene, ocimene, camphene, β-pinene, and α-pinene (Figure 1). These data are from the composition analysis performed with the Faiola laboratory TD-GC-MS. Some samples contained minor contributions from other peaks (not shown), but these six monoterpenes accounted for >95% of the total terpene signal in all samples (by integrated peak area). Limonene had the most abundant peak area in all VCP sources, contributing to 97%, 89%, 78%, and 99% of the monoterpene signal for the shampoo, rose shower gel, shower scrub, and rose body wash products, respectively. β-Pinene was a larger contributor in the rose shower gel (11%) and shower scrub (10%) than in the other two VCP sources. The two pine tree species sampled on the UCI campus were also strong limonene emitters, contributing >90% to the total integrated area for loblolly pine and 50% for slash pine. These results are consistent with previous research findings. Limonene was identified as the most prevalent compound in a study that included 249 different common consumer products.^23^ Additionally, small amounts of other monoterpenes like α-pinene and β-pinene have been observed in fragranced products.^24^ Interestingly, monoterpene emissions from citrus plants typically are not dominated by limonene to the same extent as their peel extracts, with limonene from citrus leaf emissions contributing anywhere from 2% to 38% of total terpene emissions but usually not being the dominant compound.^25,26^ This suggests that there could be very different terpene biosynthesis pathways and regulation occurring in peels versus leaves of citrus plants, and we do not necessarily expect the enantiomeric composition in the leaf emissions versus the peel extracts from citrus plants would be the same. Therefore, any results shown here for VCP limonene sources should not be interpreted to represent citrus tree emissions broadly because we do not have measurements for emissions of limonene from citrus leaves.

### 3.2 Limonene Enantiomeric Analysis

**Figure 2:**
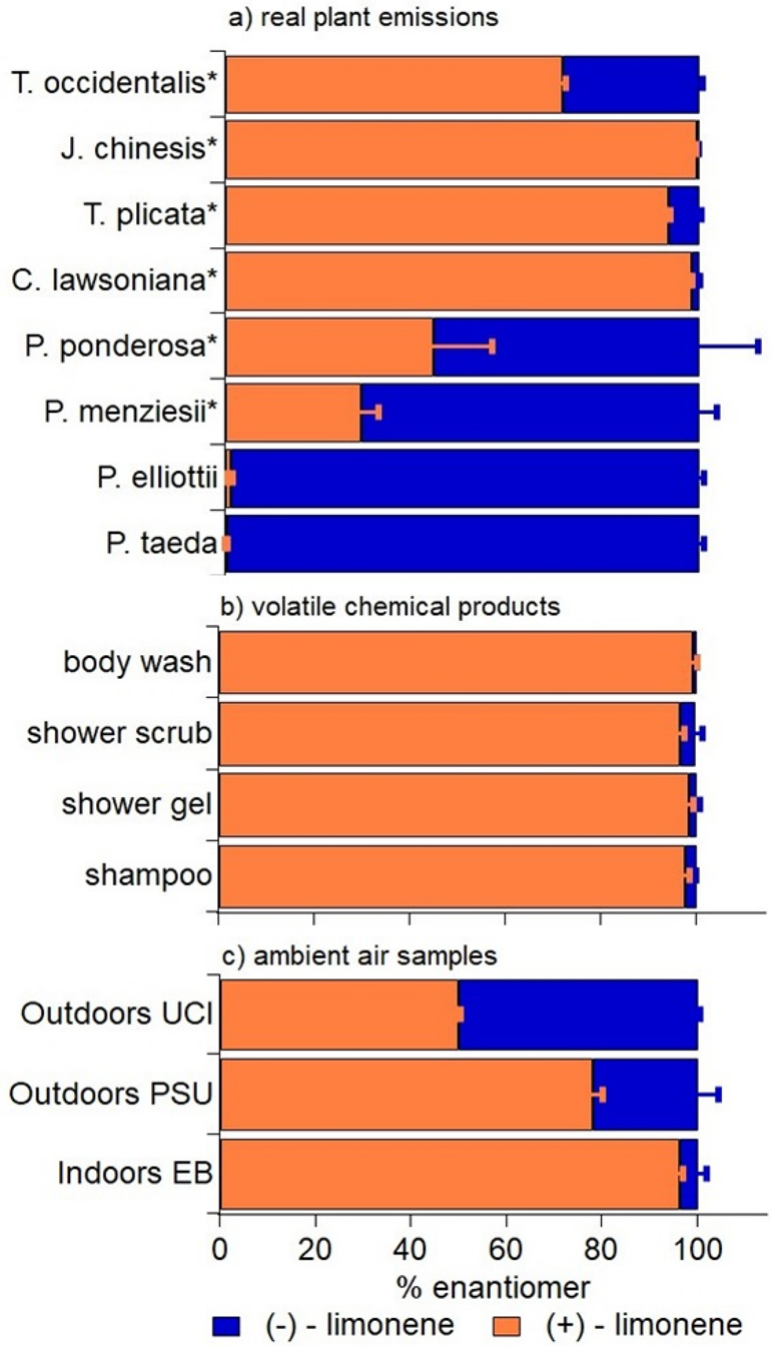
Relative abundance in percent of (−)-limonene and (+)-limonene for different samples from (a) plant emission sources, (b) fragranced VCP sources, and (c) ambient samples collected on the UCI and PSU campuses. Error bars represent either the standard deviation of multiple samples or analytical uncertainty when replicate numbers were too low to use the standard deviation. The uncertainty estimate is described in more detail in Table S2. The first six tree species in panel a are marked with an asterisk after the tree name that denotes those that were originally reported by Wang et al.^21^ and are included to provide additional context for the range of enantiomeric ratios expected from conifer emissions. The last two tree species are the tree emissions that were measured in our study. Some common names for the conifers are as follows from top to bottom: norther white-cedar, Chinese juniper, western red-cedar, Port Orford cedar, ponderosa pine, Douglas fir, slash pine, and loblolly pine, respectively. Note that common names can vary regionally, and many of these plants have multiple common names.

Limonene enantiomeric ratios were quantified for all VCP and biogenic sources included in this study (Figure 2a,b). The enantiomeric profiles were very similar among VCP sources, with (+)-limonene contributing >95% of the total limonene emissions (Figure 2b). In contrast, limonene emitted by the pine trees sampled on the UCI campus was >98% (−)-limonene (Figure 2a). Also shown in the figure are limonene enantiomeric ratios from biogenic emissions of six other conifers reported in another study for comparison.^21^ Overall, limonene from real plants is often dominated by (−)-limonene but is generally more variable than that from the different VCP sources. The dominance of (+)-limonene in VCP emission sources aligns with our prediction based on what is known about the ingredients and production processes commonly used to create these products. Essential oils extracted from orange, lemon, and lime peels are widely used in the industrial production of fragranced VCPs, and the limonene in essential oils from citrus peels is primarily the (+)-enantiomer, most often contributing >95% of the total limonene emissions.^11−14,27,28^ The (+)-limonene enantiomer is desirable in these applications because it is associated with the perceived “citrusy” smell of these consumer products.^29^ In fact, recommendations for evaluating the authenticity and purity of citrus essential oils include enantiomeric analysis to confirm that the enantiomeric ratios of monoterpenes align with the expected values from the pure essential oils, such as the (+)-limonene associated with citrus.^30^

Limonene enantiomeric ratios were quantified from three different air samples representing a range of environments (Figure 2c). An indoor air sample was collected on the PSU campus in the EB. Two outdoor air samples were collected on or near the UCI and PSU campuses: one in an urban park in Portland (Park Blocks) and one on the roof of Steinhaus Hall in Irvine. The indoor air sample was dominated by the (+)-limonene enantiomer, which would be expected in an environment more influenced by anthropogenic sources. The overall monoterpene composition is also dominated by limonene, consistent with expectations for a VCP limonene source (Figure S2).^7^ The two outdoor samples were mixtures of the enantiomers. If we assume that all (−)-limonene is derived from urban plant emissions, then we can set lower bounds for the contribution of limonene from biogenic sources as 22% and 50% in the urban park in Portland and UCI campus, respectively. This is a lower bound because (+)-limonene could be emitted by both trees and VCPs, and it is likely that at least some of the (+)-limonene is also coming from living vegetation. At the UCI sampling location, most of the tree species near the sampling location are loblolly pines (*P. taeda*) and eucalyptus. We do not have any measurements from eucalyptus, but emissions of limonene from loblolly pines are almost entirely (−)-limonene (Figure 2a). Therefore, in this location, it is possible that most of the (−)-limonene is sourced from real plant emissions and most of the (+)-limonene is sourced from fragranced VCPs. At the PSU outdoor sampling location, the surrounding trees primarily included European beech, American elm, northern red oak, and western hemlock. There have been no reports of the limonene enantiomeric ratios for these tree species, to the best of our knowledge; therefore, the 22% (−)-limonene is a lower bound estimate of the real plant emission contribution. At both locations, the monoterpene profile is dominated by α-pinene with just 5% and 12%, respectively, of the total monoterpene signal contributed by limonene. This further supports a prominent real plant emission source at both outdoor locations (Figures S1 and S2).

In summary, we demonstrated that limonene from a range of personal care products is primarily composed of the (+)-limonene enantiomer. We also illustrated that emissions from real plants can emit both limonene enantiomers, supported with our own new measurements and the results published previously by our co-authors. If these results are representative of emissions from fragranced VCPs, atmospheric measurements of (−)-limonene in urban spaces would provide a lower bound for the contribution of limonene from biogenic sources or, alternatively, the (+)-limonene would provide an upper bound for the contribution from fragranced VCPs. It is possible that some household products could have a different limonene enantiomeric fingerprint, particularly for products that use essential oils sourced from non-citrus plants, such as pines.^31^ However, citrus essential oils account for the largest proportion of commercial natural flavors and fragrances,^32^ so we believe the anthropogenic fragrance signature is likely consistent with the results shown in this study. Future studies should quantify terpene enantiomeric ratios from more household products and more locations to follow up on the results shown here. In particular, comprehensive surveys of the monoterpene enantiomeric ratios from urban trees would significantly improve our ability to use ambient enantiomer measurements for source apportionment. Others have shown that enantiomeric ratios are stable within a species across seasons and environmental contexts,^21^ so this type of survey data would improve our understanding of how much of the (+)-limonene in air is coming from living plant emissions. This would further test the potential utility of the analytical method as a useful tool for urban terpene source apportionment. It is possible that other terpene enantiomers could provide additional useful information. For example, α-pinene in pine essential oils is primarily (−)-α-pinene, but the pine essential oils sold on the market have excess (+)-α-pinene, indicating that they are often supplemented with essential oils from other sources.^33^ Importantly, the ambient measurements reported here suggest that atmospheric limonene in the urban locations we sampled is not all derived from fragrance-based VCPs, and therefore, using limonene as a tracer would lead to overestimations of fragrance-based VCP emissions. Ultimately, enantiomeric analysis can be used to complement measurements already being conducted by the air quality research community for improving the source apportionment of urban terpenes. We think it would be informative to characterize the diurnal and seasonal trends in VOC composition and enantiomeric ratios to tease apart anthropogenic versus biogenic contributions. In some locations, wintertime measurements could be used to target anthropogenic sources of limonene (as shown by Coggon et al.^34^), but that will not work as effectively in warmer locations. The diurnal variation between night and day could be an alternative approach in which it is likely that biogenic emissions are the major source at night; limonene is generally considered to have a strong light-independent component^35^ and would continue to be emitted at night when people are less active and personal care product emissions are lower.^34^ Spatial monitoring networks across a greenness gradient in the city would also be a useful measurement strategy for characterizing enantiomeric signatures across a gradient of expected biogenic influence. Finally, collecting more measurements in indoor environments would provide information about the anthropogenic enantiomeric signature. Continued monitoring of enantiomeric ratios is particularly important in a context in which biogenic VOC emissions in urban spaces are likely increasing due to urban greening programs^36^ and the increased frequency of heat waves associated with climate change.^4,5^

## Supporting Information Available

Analytical methods and uncertainty (section S1), percent contribution to the integrated area for monoterpene peaks in the UCI ambient samples (Figure S1), percent contribution to the integrated area for monoterpene peaks in the PSU ambient samples (Figure S2), summary of Faiola lab TD-GC-MS standard cartridges for four different monoterpenes (Table S1), and summary of the standard deviations for the enantiomeric analysis for all samples (Table S2) (PDF)
