# Supplementary material for: Limonene Enantiomeric Ratios from Anthropogenic and Biogenic Emission Sources
Source: Environ Sci Technol Lett. 2024 Feb 2;11(2):130–5. doi: 10.1021/acs.estlett.3c00794 (PMC10867824; doi:10.1021/acs.estlett.3c00794)
Supplement: Supplementary file 1 — ez3c00794_si_001.pdf [file ez3c00794_si_001.pdf]

Supporting Information

Limonene enantiomeric ratios from anthropogenic and biogenic emission sources

Authors: Shan Gu[1], Wentai Luo[2], Avisa Charmchi[1,3], Kevin J McWhirter[2], Todd Rosenstiel[4], James Pankow[2], Celia L. Faiola[1,3]*

[1]Ecology and Evolutionary Biology, University of California Irvine, Irvine, CA, 92697, USA

[2]Civil and Environmental Engineering, Portland State University, Portland, OR, 97201, USA

[3]Chemistry, University of California Irvine, Irvine, CA, 92697 USA

[4] Biology, Portland State University, Portland, OR, 97201, USA

*Corresponding Author

Contents:

- Section S.1: Analytical methods and uncertainty
- Figure S1. Percent contribution to integrated area for monoterpene peaks in the UC Irvine ambient samples.
- Figure S2. Percent contribution to integrated area for monoterpene peaks in the Portland State University ambient samples.
- Table S1. Summary of Faiola lab TD-GC-MS standard cartridges for four different monoterpenes
- Table S2. Summary of the standard deviations for the enantiomeric analysis for all samples.

Section S.1: Analytical methods and uncertainty

The GC method for the Faiola lab TD-GC-MS analysis was set up as follows: The helium flow rate through GC column was 1 mL min^{-1} and the GC oven temperature ramp process was 40˚C for 2 minutes, 10.0˚C min^{-1} to 90˚C and hold for 1 minute, 10˚C min^{-1} to 210˚C, 30˚C min^{-1} to 275˚C, and a final 2-minute hold. The mass of the limonene collected on the cartridge was calculated from the integrated TIC based on an instrument calibration with a limonene standard (Alfa Aesar; CAS: 5989-27-5). The emission profiles are presented in percent integrated area rather than percent by mass because we did not have authentic standards available for all the compounds observed, and we were primarily interested in quantifying the limonene signal. We ran 6 standard cartridges generated from Sigma-Aldrich chemical standards of limonene, alpha-pinene, beta-pinene, and 3-carene. A table summarizing the standard cartridge data is shown in Table S1. The relative response of alpha-pinene, beta-pinene and 3-carene to the same unit mass of limonene was 2.5 +/- 6.8%, 1.7 +/- 6.3%, and 1.9 +/- 5.6%. Uncertainties are based on the standard deviation from 6 replicate standard cartridges. The relative response factors indicate that the instrument tends to be less sensitive to limonene compared to the other monoterpenes (they are all greater than 1). Therefore, the % contribution to integrated peak area from limonene is likely an underestimate of the actual contribution to the emissions by mass.

For the enantiomeric analysis conducted at Portland State University, the cartridge samples were thermally desorbed at 250 °C for 8 minutes at 40 mL/min of helium and trapped onto a Tenax TA focusing trap at 0 °C. The trap was then thermally desorbed at 275 °C. For the VCP and plant cartridge samples, the GC column is a Beta DEX™ 120 (30m, 0.25 mm i.d, and 0.25 µm film thickness, Supelco Inc., Bellefonte, PA). The GC oven program was 55°C for 5 min, 1 °C/min to 90 °C, 3 °C/min to 105 °C, and then 20 °C/min to 220 °C. The GC was operated at constant pressure of 17 psi. For the air samples, the GC column was a CP-cyclodextrin B-2,3,6-M-19 (50m, 0.25mm id, 0.25um film thickness; Agilent Inc., Santa Clara, CA). The GC oven program was 80°C for 5 min, 1.5 °C/min to 105 °C, 15 °C/min to 160 °C, and then 20 °C/min to 220 °C and hold at 220 °C for 2 min. The GC was operated at constant pressure of 30 psi. The percentage of each limonene enantiomer was calculated by dividing the peak area of m/z 68 (the dominant limonene ion) for each enantiomer by the sum of both enantiomers' peak areas. The standards of DL limonene (CAS: 138-86-3) and (+)-limonene (CAS: 5989-27-5) were purchased from Sigma Aldrich Inc. (St. Louis, MO) at ≥ 97 % purity. The analytical uncertainty associated with the enantiomeric analysis is described in detail in Wang et al., 2022. Briefly, the variation is concentration dependent. For samples with over 10ng mass, the variation is equal to or less than 10 %. For samples containing 2 to 10ng, the variation can be as high as 20%. For samples containing less than 2 ng, the variation can be as high as 55 %. Therefore, the coefficient of variation is also related to the percent contribution of the enantiomer (shown in Figure 6 in Wang et al., 2022). For the personal care products, the variation for (+)-limonene would be very small because we had plenty of mass, but the variation for the (-)-limonene would be high due to its very low concentration. A summary of the limonene enantiomer percent contributions and associated estimated uncertainty is provided in Table S2. The instrument sensitivity is high, easily measuring just 1 ng of material. This means if one were to sample 30 liters through the

cartridge, the theoretical detection limit (at 1 ng of limonene) would be about 0.006 ppb. Sampling for 3 hours at 250 mL per minute corresponds to 45 liters of air sampled, for example, so it is possible to push the temporal resolution higher than we did in this study. However, some other major limitations to consider in this analysis include 1) interference from other ambient VOCs that could co-elute with limonene and 2) very low concentrations of one enantiomer even if there was plenty of total limonene sampled on the cartridge. Both of these challenges could make enantiomeric separation on the column difficult and potentially prevent quantitation.

Figure S1. Percent contribution to integrated area for monoterpene peaks in the UC Irvine ambient samples.

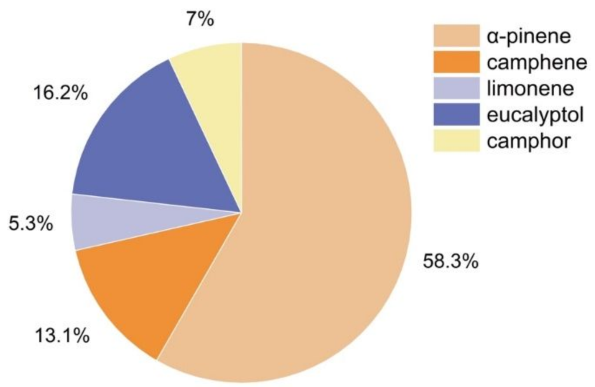

Figure S2. Percent contribution to integrated area for monoterpene peaks in the Portland State University ambient samples.

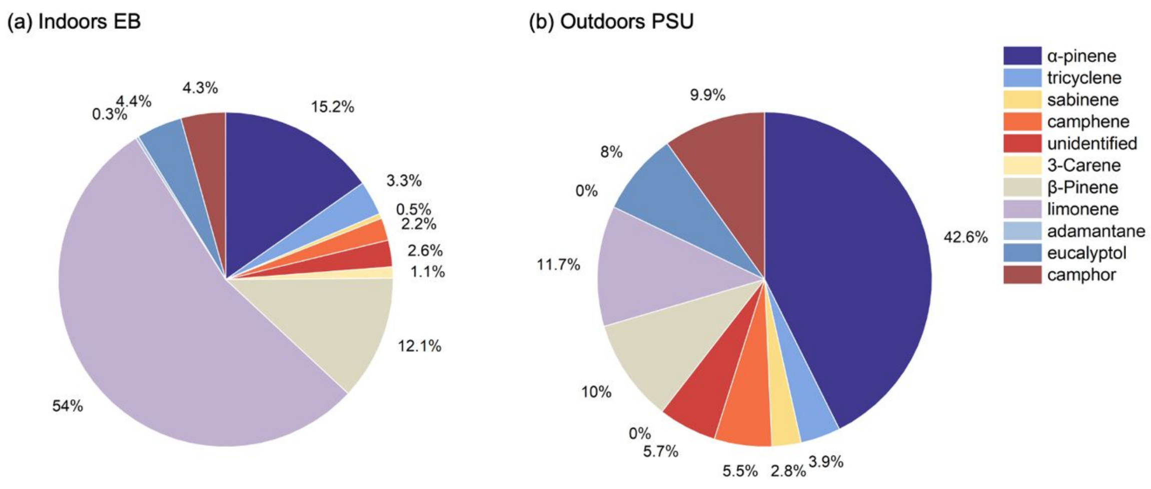

Table S1. Summary of Faiola lab TD-GC-MS standard cartridges for four different monoterpenes.

	Mass on standard cartridge (ng)	Response factor (area/ng)	Relative response factor to limonene (unitless)
Limonene	42.2	1.1e5	1 (by definition)
Alpha-pinene	42.9	2.8e5	2.5 +/- 6.8%
Beta-pinene	43.6	1.9e5	1.7 +/- 6.4%
3-carene	43.4	2.1e5	1.9 +/- 5.6%

Table S2. Summary of the enantiomer percent values and estimated uncertainty for each datapoint in Figure 2 of the main text

Sources	(+)-limonene (%)	uncertainty	(-)-limonene (%)	uncertainty
VCP				
Shampoo[a]	97.68	0.98	2.32	0.12
Shower gel[a]	98.37	0.98	1.63	0.82
Shower scrub[a]	96.62	0.96	3.38	1.69
Body wash[a]	99.20	0.99	0.80	0.40
Plant				
P. taeda[a]	0.29	0.06	99.71	0.06
P. elliottii[a]	1.16	0.21	98.84	0.21

P. menziesii[b]	28.70	3.90	71.30	3.90
P. ponderosa[b]	44.00	12.60	56.00	12.60
C. lawsoniana[b]	98.50	0.16	1.50	0.16
T. plicata[b]	93.50	0.50	6.50	0.50
J. chinesis[b]	99.58	0.02	0.42	0.02
T. occidentalis[b]	71.10	0.79	28.90	0.79
Ambient				
Indoors EB[c]	96.19	0.96	3.81	1.91
Outdoors PSU[c]	78.15	2.34	21.85	4.37
Outdoors UCI[d]	49.91	0.58	50.09	0.58

[a] Uncertainty values are provided based on the analytical uncertainty presented in Wang et al., (2022) Figure 6. This approach was used because we only had duplicate cartridges so this represents a more conservative estimate of the uncertainty than using standard deviation with such a small sample size.

[b] Enantiomeric data are from Wang et al., (2022) and uncertainty is based on the standard deviation of 6 replicate tree samples

[c] Samples collected from PSU campus without duplicates. Uncertainty values are provided based on the analytical uncertainty presented in Wang et al., (2022) Figure 6.

[d] Samples collected on UCI campus. Uncertainty is based on the standard deviation of 10 cartridges sampled across 5 different days.

REFERENCES

(1) Wang, Y.; Luo, W.; Rosenstiel, T. N.; Pankow, J. F. Measurement of Enantiomer Percentages for Five Monoterpenes from Six Conifer Species by Cartridge-Tube-Based

Passive Sampling Adsorption–Thermal Desorption (Ps-ATD). *Atmospheric Measurement Techniques* **2022**, *15* (15), 4651–4661. https://doi.org/10.5194/amt-15-4651-2022.
